# The Bacterial Amyloid Curli Is Associated with Urinary Source Bloodstream Infection

**DOI:** 10.1371/journal.pone.0086009

**Published:** 2014-01-20

**Authors:** Chia Hung, Jonas Marschall, Carey-Ann D. Burnham, Albert S. Byun, Jeffrey P. Henderson

**Affiliations:** 1 Division of Infectious Diseases, Department of Medicine, Washington University School of Medicine, St. Louis, Missouri, United States of America; 2 Center for Women’s Infectious Diseases Research, Washington University School of Medicine, St. Louis, Missouri, United States of America; 3 Department of Infectious Diseases, Bern University Hospital, Bern, Switzerland; 4 Department of Pathology & Immunology, Washington University School of Medicine, St. Louis, Missouri, United States of America; 5 Department of Pediatrics, Washington University School of Medicine, St. Louis, Missouri, United States of America; Indian Institute of Science, India

## Abstract

Urinary tract infections are the most common cause of *E. coli* bloodstream infections (BSI) but the mechanism of bloodstream invasion is poorly understood. Some clinical isolates have been observed to shield themselves with extracellular amyloid fibers called curli at physiologic temperature. We hypothesize that curli fiber assembly at 37°C promotes bacteremic progression by urinary *E. coli* strains. Curli expression by cultured *E. coli* isolates from bacteriuric patients in the presence and absence of bacteremia were compared using Western blotting following amyloid fiber disruption with hexafluoroisopropanol. At 37°C, urinary isolates from bacteremic patients were more likely to express curli than those from non-bacteremic patients [16/22 (73%) vs. 7/21 (33%); *p* = 0.01]. No significant difference in curli expression was observed at 30°C [86% (19/22) vs. 76% (16/21); *p* = 0.5]. Isolates were clonally diverse between patients, indicating that this phenotype is distributed across multiple lineages. Most same-patient urine and blood isolates were highly related, consistent with direct invasion of urinary bacteria into the bloodstream. 37°C curli expression was associated with bacteremic progression of urinary *E. coli* isolates in this population. These findings suggest new future diagnostic and virulence-targeting therapeutic approaches.

## Introduction

Curli fibers are extracellular amyloid fibrils that are variably expressed by *E. coli* (for review, see [1]). Characteristic of amyloids, curli fibers are highly stable, insoluble, high molecular weight protein complexes dominated by a beta sheet secondary structure. While many amyloid fibers have been described for different bacterial organisms, curli is the only known amyloid fibers encoded by *E. coli* and other Enterobactericiae such as *Salmonella* spp. (for review, see [2]). Unlike human amyloids, curli fibers are deliberately assembled by dedicated bacterial machinery [3]–[6]. The curli fiber biogenesis requires both structural (CsgA and CsgB) and non-structural (CsgD, CsgE, CsgF, and CsgG) components encoded by genes on two divergent operons [4], [5], [7], [8]. Curli assembly follows an ordered process termed “nucleation-precipitation” that has been extensively studied in many laboratories (for review, please see [1]). Curli fibers are composed of primarily CsgA proteins with CsgB proteins as minor components. During curli assembly, CsgB monomers are exported outside of bacteria through CsgG pores, fold into proper conformation, and associate with bacterial cell surface [7]. Chaperoned by CsgE proteins, CsgA monomers are also exported in the same fashion as unfolded proteins out to the cell surfaces. Out on bacterial surfaces, initially exported CsgA monomoers fold into proper conformation upon interaction with CsgB and associate with CsgB proteins, forming nucleation centers. Subsequent CsgA monomers exported out onto bacterial surfaces quickly assume the proper conformation upon interaction with the nucleation centers and are incorporated onto the growing fibers in association with the existing CsgA proteins in the fibers. Curli fibers have been implicated in biofilm formation on both abiotic and biotic surfaces [9]–[12], persistent avian colibacillosis [13], and immune modulation in mammalian hosts [14]. Curli fibers also have been implicated to play a role in bladder colonization at 6 hours post-infection in an experimental UTI model in mice [9]. In that report, deletion of *csgA* gene in a prototypic uropathogenic *E. coli* resulted in reduced bladder colonizations at 6 hrs post-infection. Based on these findings, curli fibers have been proposed to be a virulence factor in human urinary tract infections (UTIs) [15] and bacteremia [16].

Upon their discovery, curli fibers were known to be expressed at temperatures below 26°C, leading to speculation that they are an adaption for survival at lower temperatures [17]. Bian *et al.* later demonstrated robust curli production at 37°C in a series of *E. coli* blood isolates from hospitalized patients [16]. Together with a demonstrated serological response to curli in septic patients, this raised the possibility that curli expression at physiologic temperature is an *E. coli* virulence trait. Whether 37°C curli production facilitates bacterial migration from the urinary tract into the bloodstream or ensures survival in the bloodstream has been unclear.

We hypothesized that curli expression by *E. coli* at physiologic temperature promotes bacteremic progression during urinary tract infections. Previous studies lacked either clear information on the clinical severity of UTI patients [18] or a non-bacteremic comparator group necessary to seek associations between curli expression and bacteremic progression [16]. To test our hypothesis, we compared curli expression between bacteremic and non-bacteremic urinary *E. coli* isolates from a prospective cohort study of hospitalized patients with urinary tract infection. Curli expression by cultured isolates was assessed with an optimized Western blot analysis. Our results revealed a strong correlation between curli expression at 37°C and urinary-source bloodstream infections. Genetic typing showed that curli expression among bacteremic isolates was distributed across multiple lineages.

## Materials and Methods

### Clinical *E. coli* Isolates and Patient Data

Clinical *E. coli* isolates were obtained through an observational study on risk factors for urinary-source bacteremia in patients with *E. coli* bacteriuria. Urine and blood isolates (if the patient was bacteremic) of enrolled patients were identified in the Barnes-Jewish Hospital Medical Microbiology Laboratory using conventional biochemical methods and stored in skim milk at −80°C [19].

### Curli Expression Analysis

Curli expression was identified by Western blotting of cultured bacteria. Bacterial isolates were patched onto Luria-Bertani (LB) plates from frozen stocks and incubated overnight at 37°C. Subsequently, a small amount of bacteria was resuspended in 100 uL of YESCA broth (10 g/L casamino acid and 1.2 g/L yeast extract) and streaked onto YESCA plates (YESCA broth supplemented with 20 g/L Bacto agar) to allow for curli expression. These plates were then incubated for 48 hours at 30°C and 37°C. For Western blotting, a 1 cm square patch of bacteria was scraped off for each strain and resuspended in 150 µL of phosphate buffered saline (PBS) (137 mM NaCl, 2.7 mM KCl, 4.3 mM Na_2_HPO_4_, 1.47 mM KH_2_PO_4_). These bacteria were pelleted by centrifugation at 14,000 rpm for 10 minutes and resuspended in 100 µL of the curli-depolymerizing agent hexafluoroisopropanol (HFIP). After 10 minutes of incubation at 23°C, samples were dried in a Savant Speedvac vacuum concentrator (ThermoFisher Scientific, Waltham, MA).

Dried HFIP-treated pellets were resuspended in 100 µL of 4× gel loading buffer (0.125 M Tris base, 10% 2-mercaptoethanol, 20% glycerol, 6% sodium dodecyl sulphate, 0.02% bromophenol blue) and boiled at 95°C for 10 minutes before loading onto SDS-PAGE gels. Ten (10) uL of each sample was loaded into the gels along with BLUEstain 3 Protein Ladder (Gold Biotechnology, St. Louis, MO) and electrophoresed at a constant current of 30 mAh. After electrophoresis, gels and PVDF membranes (pre-wetted in methanol) were equilibrated in Laemmli transfer buffer (14.4 g/L glycine, 3.02 g/L Tris base, 10% methanol) for 15 minutes. Western transfer of proteins onto PVDF membranes was carried out at 30 V and 4°C overnight.

Following transfer, PVDF membranes were rinsed in TBST (50 mM Tris, 150 mM NaCl, 0.05% Tween 20, pH 7.6) and placed in blocking buffer (TBST, 1.5% BSA, 1.5% non-fat dry milk) for 1 hour. Membranes were immunoblotted with rabbit anti-CsgA antisera (Proteintech, Chicago, IL) in the blocking buffer (1∶5,000 dilution) for 1 hour. After four 5-minute TBST wash cycles, membranes were incubated with horseradish peroxidase (HRP)-conjugated goat-anti-rabbit secondary antibody in the blocking buffer (1∶5,000 dilution) for 1 hour. After 4 wash cycles with TBST, the membranes were developed with SuperSignal West Pico Chemiluminescent Substrate (Thermo Scientific, Rockford, IL) for 5 minutes and then immediately exposed to radiography film in the darkroom. Bacteria expressing curli fibers produced a 15 kDa reactive band on the radiography films corresponding to CsgA, the major structural protein of curli fibers.

### Enterobacterial Repetitive Intergenic Consensus-PCR Fingerprinting

The relatedness of bacterial isolates was assessed using Enterobacterial Repetitive Intergenic Consensus (ERIC)-PCR in conjunction with the DiversiLab system [20]. In brief, DNA was extracted from isolates using the MoBio Bacteremia DNA Isolation Kit (MoBio Laboratories, Carlsbad, CA) and ERIC-PCR was performed using RAPD Ready-to-go analysis beads (GE Healthcare, Pittsburg, PA) in a final reaction volume of 25 uL, including approximately 100 ng of genomic DNA, and 125 pmol of each primer (ERIC1: ATGTAAGCTCCTGGGGATTCAC and ERIC 2: AAGTAAGTGACTGGGGTGAGCG). Amplification cycling parameters included 40 cycles of: 94°C for 1 min, 54°C for 1 min and 72°C for 2 min, followed by a final extension step of 72°C for 5 minutes. Resulting PCR products were resolved using DiversiLab DNA chips (bioMerieux, Durham, NC) on the Agilent 2100 system (Agilent Technologies, Santa Cara, CA). The DiversiLab software was used to compare banding patterns and determine the similarity of the isolates (similarity index, SI). The SI of 85% is the standard cut-off level for related isolates of Gram-negative bacteria in an outbreak investigation or epidemiology purpose [21]–[24].

### Statistical Analysis

Statistical analyses were done with the software package PRISM (GraphPad, La Jolla CA) and with EpiInfo (CDC, Atlanta GA). We used *Chi*-square test or Fisher’s exact test as appropriate to compare rates between groups. A *p* value of <0.05 was considered statistically significant.

### Ethics Statement

We obtained bacterial isolates and the corresponding clinical information from a previously described [19] observational study in which patients admitted to Barnes-Jewish Hospital were enrolled. The Human Research Protection Office at Washington University (which acts as the Institutional Review Board for Barnes-Jewish Hospital) approved the study with a waiver of informed consent due to its observational nature [Approval# 201105193]. All bacterial isolates and clinical information (used to determine the severity of urinary tract infection) were de-identified prior to the analyses presented here.

## Results

### Patient Isolates

To determine whether curli fiber expression is associated with urinary-source bacteremia, we compared bacterial isolates from 22 bacteremic patients to 21 non-bacteremic urinary isolate controls [19]. More bacteremic than non-bacteremic patients exhibited fever [73% (16/22) vs. 48% (10/21)] or septicemia [91% (20/22) vs. 67% (14/21)]; however, the frequency of these clinical symptoms between the two groups were not statistically significant (*p* = 0.08 and *p* = 0.06, respectively). Pyelonephritis was more frequent in bacteremic than in non-bacteremic patients [77% (17/22) vs. 48% (10/21); *p* = 0.04].

### Curli Expression as a Function of Temperature and UTI Severity

To determine whether curli expression at 37°C is associated with bloodstream infections, we compared cultured bacteremic and non-bacteremic urine isolates by Western blot detection of CsgA, the major curli subunit. The percentage of curli expressors was significantly higher in bacteremic versus non-bacteremic isolates [73% (16/22) vs. 33% (7/21) respectively; *p* = 0.01] (Table 1). To assess whether this difference reflects an intrinsic curli-deficiency (in some strains) or a specific temperature effect, we compared curli expression between these strains at 30°C. At 30°C, curli expression frequencies were no longer different between bacteremic versus non-bacteremic urinary isolates [86% (19/22) vs. 76% (16/21); *p* = 0.5]. Overall, most strains were capable of curli expression, with a higher proportion of all urine isolates expressing curli at 30°C [35/43 (81%)] than at 37°C [23/43 (55%)] (*p* = 0.01).

**Table 1:** Curli fiber expression in urinary *E. coli* specimens from bacteremic versus non-bacteremic patients.

	30°C			37°C		
	BacteremicUrine Isolates	Non-bacteremic UrineIsolates (controls)	*p* value	Bacteremic Urine Isolates	Non-bacteremic UrineIsolates (controls)	*p* value
**Positive**	19 (86%)	16 (76%)	0.5	16 (73%)	7 (33%)	0.01
**Total**	22	21		22	21	

### Genetic Diversity of Bacteremic Isolates

**Figure 1:**
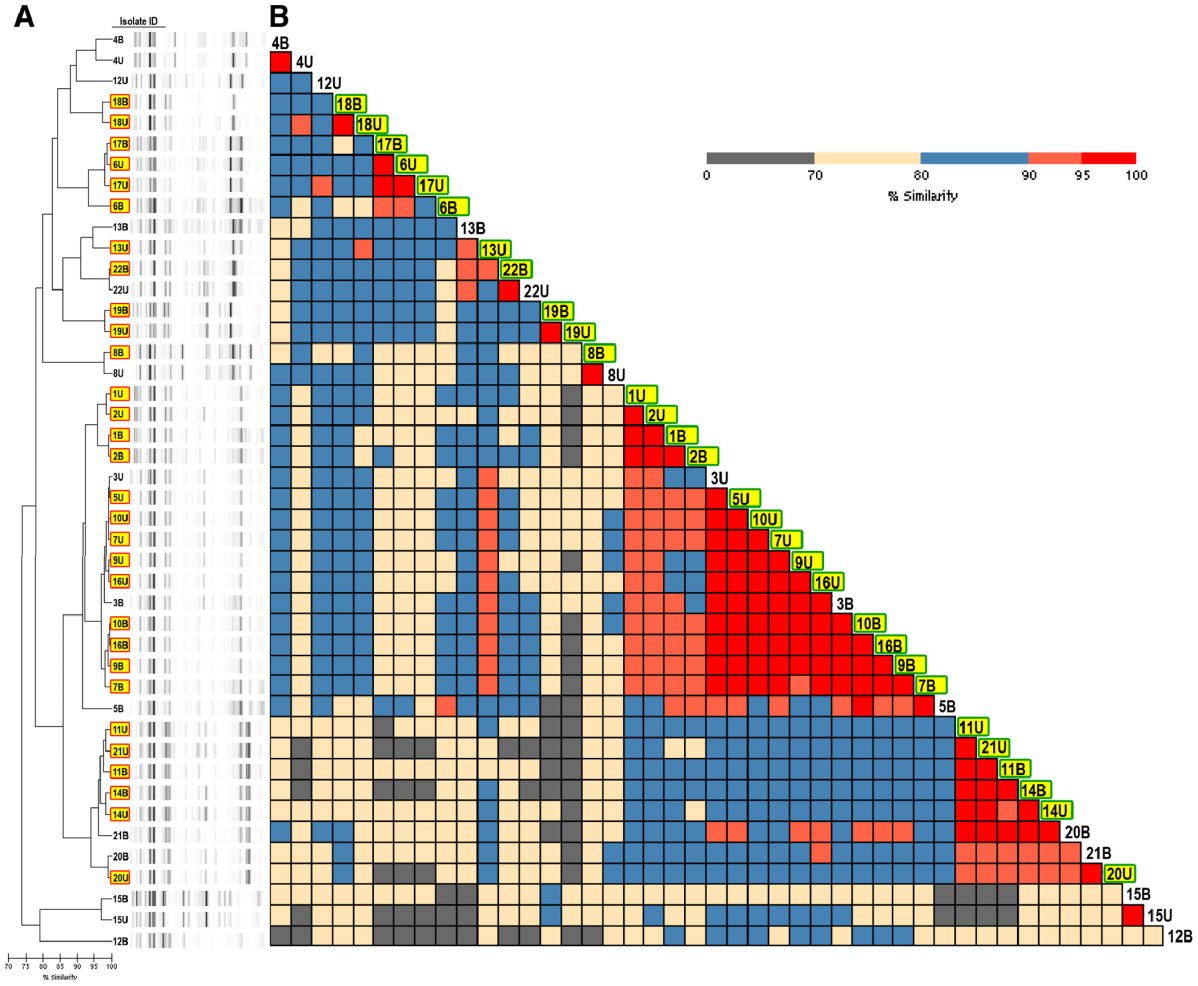
ERIC-PCR analysis of bacterial isolates from bacteremic patients. Isolates are labeled by patient number followed by “B” for blood isolates and “U” for urine isolates. (A) Dendrogram analysis of bacterial isolates from bacteremic patients. Bacterial isolates from bacteremic patients were characterized by ERIC-PCR using DiversiLab software to assess strain relatedness. Dendrogram analysis revealed marked genetic similarities between blood and urine isolates of the same patients with clustering to the same sub-branches or nodes. However, bacterial isolates expressing curli at 37°C (highlighted in yellow) were distributed widely across multiple ERIC groups. (B) Inter- and intra-patient isolate genetic similarity matrix. A genetic similarity matrix based on ERIC-PCR banding patterns was generated for bacterial isolates from bacteremic patients. The data in the dendrogram in (A) is lined up with that in the similarity matrix (B) according to the isolates. With the exception of isolates from patient 12, intra-patient urinary and blood isolate similarities exceed 90%. Isolate similarity between patients was lower, with only a small cluster of isolates from 5 patients sharing over 95% similarity. As with the dendrogram, isolates expressing curli at 37°C (highlighted in yellow) were dispersed throughout the matrix.

To determine whether curli expression reflects the idiosyncratic property of a single clonal epidemic strain, rather than an independent pathogenic factor, we used ERIC-PCR to evaluate bacteremic strain relatedness (Figure 1A). For Enterobacteriaceae, this ERIC-PCR assay is similar to PFGE in its ability to discriminate between related isolates and to group like isolates as similar [21], [22], [25]. 37°C curli-expressers were distributed across a range of ERIC types and include multiple pairs with less than the 85% similarity cutoff often used to distinguish strains (Figure 1B). Overall, this analysis shows that the curli expression phenotype observed in this cohort was not attributable to a single epidemic strain.

### Relationship of Same-patient Urine and Blood Isolates

In UTI-associated bloodstream infection, the dominant urinary *E. coli* strain may directly invade the bloodstream or instead facilitate invasion by a second *E. coli* strain in the context of a multi-strain infection. To address these possibilities, we determined the relatedness of same-patient blood and urinary isolates using both ERIC-PCR and curli expression. Among the bacterial isolate pairs from 22 patients analyzed, 16 pairs showed ≥95% genetic relatedness while 5 pairs were between 90% and 95% in genetic relatedness, which were above the standard 85% similarity cutoff (Table 2). Only the non-curli-expressing blood and urine isolate pair from patient #12 had a similarity index suggesting that the two strains were not related (SI <85%). A similarly high degree of urine-blood pair relatedness has also been observed in a PFGE study of a separate patient cohort [26]. For curli expression, no significant difference was noted between blood vs. urine isolates within bacteremic individuals at either 37°C [59% (13/22) vs. 73% (16/22); *p* = 0.5] or 30°C [77% (17/22) vs. 86% (19/22); *p* = 0.7]. Altogether, these findings support, but do not prove, that the dominant urinary isolate is most likely to become the blood isolate in *E. coli* bacteremia patients. A more detailed molecular typing effort might distinguish more unrelated pairs.

**Table 2:** 37°C curli expression and ERIC-PCR similarity between same-patient blood and urine isolates.

Patient	Blood Isolate	Urine Isolate	Similarity Index
1	+	+	95
2	+	+	95
3	−	−	95
4	−	−	95
5	−	+	90
6	+	+	90
7	+	+	95
8	+	−	95
9	+	+	95
10	+	+	95
11	+	+	95
12	−	−	70
13	−	+	90
14	+	+	95
15	−	−	95
16	+	+	95
17	+	+	95
18	+	+	95
19	+	+	95
20	−	+	90
21	−	+	90
22	+	−	95

## Discussion

In this study of *E. coli* isolates, we observed a correlation between bacterial curli fiber expression at 37°C and secondary *E. coli* bloodstream infection. ERIC-PCR typing showed that blood *E. coli* isolates almost always match the corresponding urine isolate. Curli expression was distributed across a genetically diverse group of urine and blood strains from bacteremic patients. Together these findings support an independent role for curli expression as a bacteremic progression correlate in urinary *E. coli* isolates.

Previous studies have shown that a substantial proportion of urinary tract [15] and bloodstream *E. coli* isolates [16] can express curli fibers. Another group, Bokranz and colleagues, studied curli fiber expression in gastrointestinal and urinary isolates at different temperatures but did not provide information on clinical severity in UTI patients [18]. It remained unclear from these studies whether curli expression is associated with bacteremia or simply reflects its association with UTI, a common source for bacteremia. The expression difference we observed in bacteremic versus non-bacteremic urinary *E. coli* isolates supports 37°C curli expression as a marker of UTI strains with bacteremic potential, alongside *kpsM* and P-related fimbrial genes [19].

Many previous studies suggested that the underlying pathophysiologic gains-of-function are attributable to curli. Kai-Larsen and colleagues have reported that curli and cellulose expression was associated with increased uropathogenic *E. coli* virulence in mouse UTI models [14]. Curli expression facilitated epithelial cell adherence and increased resistance to the human antimicrobial peptide LL-37, urinary levels of which are increased during UTI [27]. Uropathogens may also use curli fibers to prepare an external peptidoglycan matrix in biofilm-related infections; a study in Shiga toxin-producing *E. coli* described how such a curli-mediated biofilm protected bacteria against environmental stress [28]. Curli’s association with some biofilm types further raise the possibility that its expression may facilitate phenotypic resistance to antibiotics commonly used for UTI. Although high relatedness between the urinary and blood isolates in each patient suggests that curli may protect expressing bacteria, the possibility remains that a curli-expressing strain may facilitate bacteremic invasion by an adjacent, non-expressing strain by shielding it in a biofilm or through an immunologic effect. These or other unidentified curli-associated properties may facilitate bacteremic progression of urinary *E. coli* strains through increased kidney or vascular invasion.

A notable limitation of our study was the heterogeneity of the control group, which consisted of patients with confirmed negative blood cultures in the setting of asymptomatic bacteriuria, cystitis, and pyelonephritis. The time-consuming Western blot-based analytical approach used in this study limited the control group sample size and remains a challenge for future work. Specific detection of curli subunits following chemical amyloid disruption, although time consuming and technically tedious, remains the gold standard method for curli detection. Because the CsgA protein comprises the vast majority of curli fibers, it is the most sensitive and specific measure of curli fiber formation. Agar plate colony morphology combined with an amyloid-binding dye - most commonly Congo Red - has also been used in published studies to detect curli expression [29]–[31]. Unfortunately, Congo Red also binds to cellulose polymers expressed by many pathogenic *E. coli* to give a signal in the absence of curli expression [29], [32], compromising the assay’s specificity. We attempted to improve this assay by combining Congo Red and Bromophenol Blue dyes (Figure S1) and comparing the results to Western blot. Unfortunately, we were unable to identify conditions that permitted clear visual interpretation of results, particularly at 37°C (Figure S2). Our findings show that Western Blot analysis, although laborious, remains as the most specific and sensitive assay to detect 37°C curli expression. Future study and application of this phenotype would benefit from a higher throughput detection method.

A genetic test for the ability to produce curli fiber at physiologic temperature would greatly facilitate testing, although it is unknown if there is a genetic marker for 37°C curli expression. While curli genes are conserved in *E. coli*, genetic factors permitting their expression at 37°C have not been described [33]. Curli expression is influenced by the regulator proteins CsgD and Crl [34]–[36], but additional regulatory inputs are possible. A wide variety of temperature-sensitive genes have been previously described and may interact with this network through protein-protein interactions, second messengers, or altered metabolism. Invertible genetic elements may also influence curli expression as they do for numerous other bacterial fibers [37]. More extensive molecular studies of this phenotype are necessary to identify its molecular basis.

In summary, these results suggest that 37°C curli expression could be used to risk-stratify patients or trigger future administration of anti-virulence therapeutics [9]. The findings reported here provide a rationale for a rapid curli-expression test that could be used alone or in combination with other bacterial markers to assess bacteremia risk in UTI patients, which could greatly impact disease management and outcomes.

## Supporting Information

Figure S1 **Morphology and phenotypic examination of prototypic UPEC and isogenic mutants grown on Congo Red/Bromophenol Blue containing YESCA plates.** Two UPEC strains (UTI89 and CFT073) isogenic mutants of UTI89 (UTI89Δ*yhjO*, UTI89Δ*csgA*, and LSR34), and a prototypic K12 *E. coli*, MG1655 were grown on YESCA plates containing Congo Red and Bromophenol Blue dyes for 48 hrs at indicated temperatures. These strains showed different colony morphology and color due to the differential expressions of curli (CU) and cellulose (CE). The genotype status of curli operon (*csg*) and cellulose synthetase (*yhjO*) is also indicated. “+” denotes the presence and “−“ denotes the absence of either genotype or phenotype.
(TIFF)

Figure S2 **Morphology and phenotypic determination of UPEC grown on Congo Red/Bromophenol Blue containing YESCA plates.** Bacteria were grown on YESCA plates containing Congo Red and Bromophenol Blue dyes to assess their ability to express curli at indicated temperatures. (A) Clinical strains isolated from either blood (b) or urine (u) of bacteremic patients were grown for 48 hrs at indicated temperatures. The curli-expression status as determined by Western blotting is indicated below each image. (B) Clinical strains isolated from the urine of UTI patients were grown for 48 hrs at indicated temperatures. The curli-expression status as determined by Western blotting is indicated below each image. Results revealed the difficulty and subjectivity of the dye-stain method in determining curli expression status in clinical isolates.
(TIFF)
