# Supplementary figures and images for: The Bacterial Amyloid Curli Is Associated with Urinary Source Bloodstream Infection
Source: PLoS One. 2014 Jan 20;9(1):e86009. doi: 10.1371/journal.pone.0086009 (PMC3896446; doi:10.1371/journal.pone.0086009)

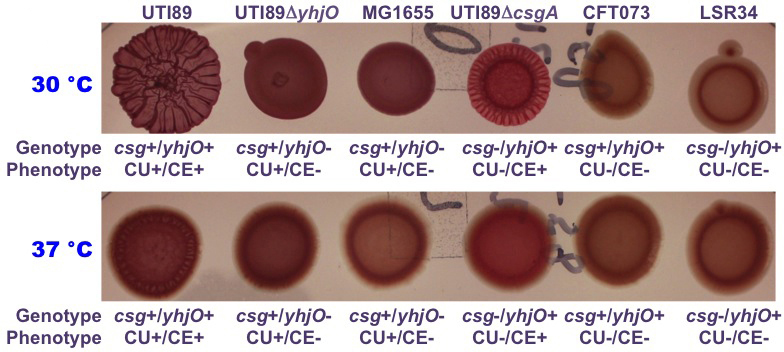

Supplement: Figure S1 — Morphology and phenotypic examination of prototypic UPEC and isogenic mutants grown on Congo Red/Bromophenol Blue containing YESCA plates. Two UPEC strains (UTI89 and CFT073) isogenic mutants of UTI89 (UTI89ΔyhjO, UTI89ΔcsgA, and LSR34), and a prototypic K12 E. coli, MG1655 were grown on YESCA plates containing Congo Red and Bromophenol Blue dyes for 48 hrs at indicated temperatures. These strains showed different colony morphology and color due to the differential expressions of curli (CU) and cellulose (CE). The genotype status of curli operon (csg) and cellulose synthetase (yhjO) is also indicated. “+” denotes the presence and “−“ denotes the absence of either genotype or phenotype. (TIFF) [file pone.0086009.s001.tiff]

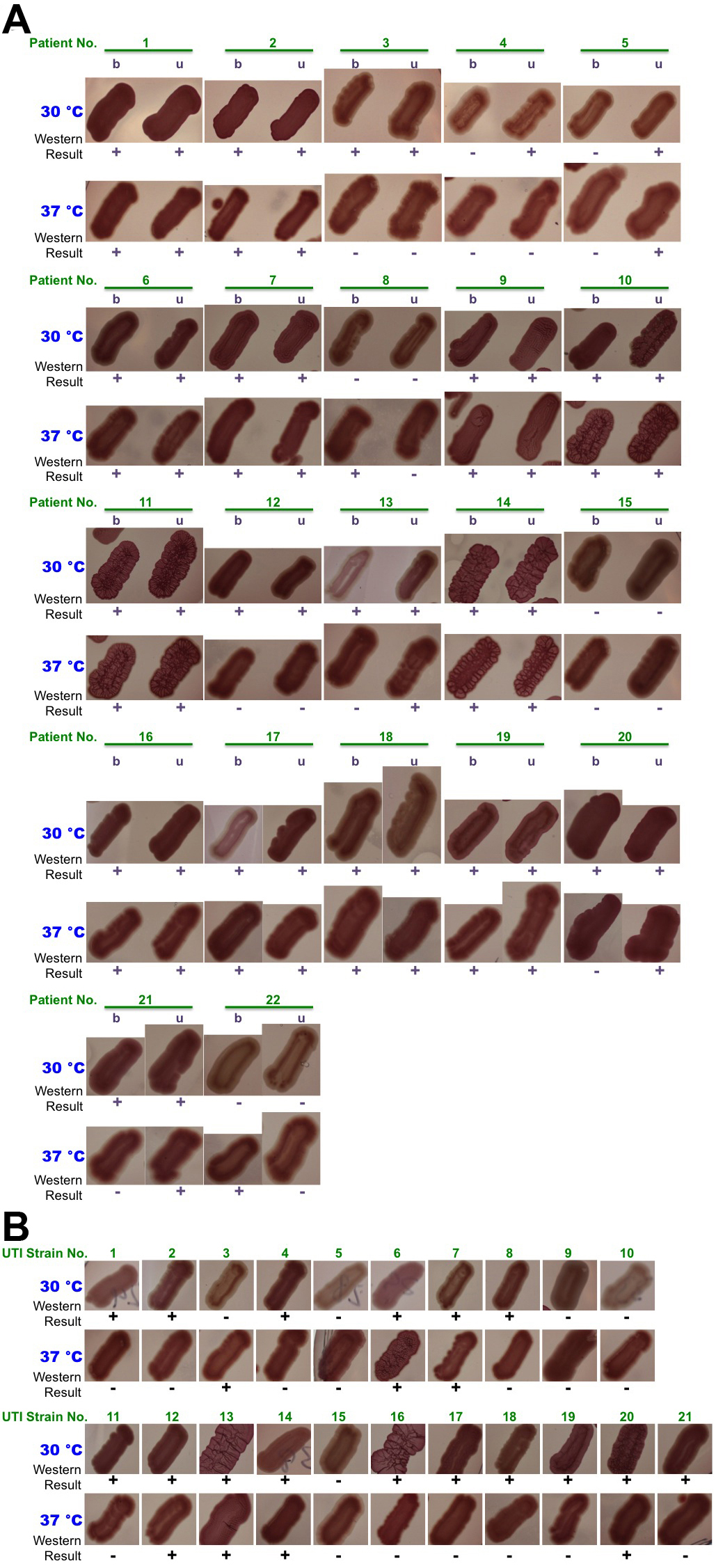

Supplement: Figure S2 — Morphology and phenotypic determination of UPEC grown on Congo Red/Bromophenol Blue containing YESCA plates. Bacteria were grown on YESCA plates containing Congo Red and Bromophenol Blue dyes to assess their ability to express curli at indicated temperatures. (A) Clinical strains isolated from either blood (b) or urine (u) of bacteremic patients were grown for 48 hrs at indicated temperatures. The curli-expression status as determined by Western blotting is indicated below each image. (B) Clinical strains isolated from the urine of UTI patients were grown for 48 hrs at indicated temperatures. The curli-expression status as determined by Western blotting is indicated below each image. Results revealed the difficulty and subjectivity of the dye-stain method in determining curli expression status in clinical isolates. (TIFF) [file pone.0086009.s002.tiff]
